# Different patterns of evolution for duplicated DNA repair genes in bacteria of the Xanthomonadales group

**DOI:** 10.1186/1471-2148-4-29

**Published:** 2004-08-27

**Authors:** Marinalva Martins-Pinheiro, Rodrigo S Galhardo, Claudia Lage, Keronninn M Lima-Bessa, Karina A Aires, Carlos FM Menck

**Affiliations:** 1 Department of Microbiology, Institute of Biomedical Sciences, Av. Prof. Lineu Prestes 1374, São Paulo, 05508-900, SP, Brazil; 2 Laboratório de Radiobiologia Molecular, Instituto de Biofísica Carlos Chagas Filho, Bloco G, Centro de Ciências da Saúde, Universidade Federal do Rio de Janeiro, 21949-900, Rio de Janeiro, RJ, Brazil

**Keywords:** DNA repair, evolution, horizontal transfer, paralogs.

## Abstract

**Background:**

DNA repair genes encode proteins that protect organisms against genetic damage generated by environmental agents and by-products of cell metabolism. The importance of these genes in life maintenance is supported by their high conservation, and the presence of duplications of such genes may be easily traced, especially in prokaryotic genomes.

**Results:**

The genome sequences of two *Xanthomonas *species were used as the basis for phylogenetic analyses of genes related to DNA repair that were found duplicated. Although 16S rRNA phylogenetic analyses confirm their classification at the basis of the gamma proteobacteria subdivision, differences were found in the origin of the various genes investigated. Except for *lexA*, detected as a recent duplication, most of the genes in more than one copy are represented by two highly divergent orthologs. Basically, one of such duplications is frequently positioned close to other gamma proteobacteria, but the second is often positioned close to unrelated bacteria. These orthologs may have occurred from old duplication events, followed by extensive gene loss, or were originated from lateral gene transfer (LGT), as is the case of the *uvrD *homolog.

**Conclusions:**

Duplications of DNA repair related genes may result in redundancy and also improve the organisms' responses to environmental challenges. Most of such duplications, in *Xanthomonas*, seem to have arisen from old events and possibly enlarge both functional and evolutionary genome potentiality.

## Background

The availability of complete genome sequences from different organisms makes it possible to identify, by similarity, potential homologs of genes that have been experimentally tested in other living beings, resulting in the recognition of putative functions for the proteins encoded by them. This research concept represents a revolutionary tool in modern biology, as the data generated allow for recognition of the presence or absence of genes, giving indications of the metabolic pathways present in such organisms and revealing possible particularities of the individuals in their natural habitat. Moreover, the possibility of obtaining gene sequences from organisms that have long diverged makes it feasible to use these data to trace their evolutionary origin [1]. Computer analysis of genome data, and its capacity to rapidly generate relevant information, contributes to a better understanding of the different evolutionary histories, especially within prokaryotes. The inferred relationship among organisms, as first defined by the use of 16S rRNA sequences, was later confirmed either by the utilization of many other conserved genes [2,3] or even by alternative strategies to trace evolution with genetic data [4]. However, the utilization of a single gene to describe the organism evolution has been contested due to genomic complexity. In fact, the accumulated data have brought evidence to sustain that many prokaryotic genes do not follow vertical transmission, revealing the occurrence of gene exchange among different species, a phenomenon known as horizontal or lateral gene transfer (LGT, reviewed by Ochman [5]).

All known forms of life present efficient systems to maintain the integrity of their genetic material. As DNA is under constant attack by different environmental agents and metabolic by-products, evolution has provided organisms with several DNA repair pathways to remove or to tolerate lesions in their genetic material. In fact, these pathways have at least two important contrasting roles in evolution, safeguarding the genome, and allowing for a certain level of mutations in the course of evolution. The critical balance of these two activities is probably the best reason for the high levels of conservation observed in DNA repair related proteins, even across the three kingdoms, Bacteria, Archaea and Eukarya. Detailed studies of many of the different DNA repair genes and protein domains have been described previously [6,7], confirming that information on DNA repair genes may be very useful as a source to track genome evolution.

In this work, we investigated the DNA repair genes in the recently described genomes from the Xanthomonadales group [8,9] following an evolutionary perspective. The Xanthomonadales group bacteria have a great economic impact on agriculture in Brazil and worldwide. The characterization of DNA repair genes from these phytopathogens could help to understand the mechanisms by which these organisms respond to environmental conditions, including plant infection. On the other hand, this could contribute to defining universal features relating DNA repair with this life style. By comparing the evolutionarily close genomes of *Xanthomonas axonopodis*, *X. campestris *and *Xylella fastidiosa*, we found that certain DNA repair genes present duplications in both *Xanthomonas *species. The great relevance of gene duplication for expanding gene families, as well as for gene innovation, is a consensus among researchers. New genes can facilitate survival in new environments or make possible the use of new metabolites. We investigated the phylogeny of such duplicated genes, in search of evidence on the mechanisms by which cells evolve their DNA repair machinery. Although most DNA repair genes follow the conserved vertical transmission found for 16S rRNA phylogeny, the data clearly show that duplications may have arisen by different means. Examples of a recent duplication, as well as of other old or LGT events, generating paralogs, are presented. These results also help in tracing the origins of LGT events that lead to redundancy and also to functional diversification, especially of *Xanthomonas *bacteria.

## Results and Discussion

DNA repair-related genes are normally highly conserved, and grant good genetic information for investigating the evolution of organisms. Orthologs of known bacterial DNA repair proteins were identified in the plant pathogen bacteria of the Xanthomonadales group and are listed in Table 1. The different gene content of these closely related bacterial species are of particular interest, given that they can reveal gene loss or acquisition as a consequence of their different lifestyles. Few genes are missing in the genome of *X. fastidiosa*, when compared to both *Xanthomonas *species: *ada-alkA *(fusion), *tag*, *phr*, *dinB *and *ligB*. For evolution purposes, however, the presence of several duplications, especially in the *Xanthomonas *genomes, will be focused here.

**Table 1:** Distribution of DNA repair genes in Xanthomonadales: presence of duplications.

**Repair Pathway**	***Xanthomonas axonopodis ***[5.27 Mbp]	***Xanthomonas campestris ***[5.08 Mbp]	***Xylella fastidiosa ***[2.73 Mbp]
**Nucleotide Excision Repair (NER)**	***uvrA(2)***, *uvrB*, ***uvrC(2)***, ***uvrD(2)***, *mfd*	***uvrA(2)***, *uvrB*, ***uvrC(2)***, *uvrD*, *mfd*	*uvrA*, *uvrB*, *uvrC*, *uvrD*, *mfd*
**Base Excision Repair (BER)**	*alkA*^a^, *fpg*, *mag*, *mutY*, *nth*, *tag*, *ung*, ***xthA (2)***	*alkA*^a^, *fpg*, *mag*, *mutY*, *nth*, *tag*, *ung*, ***xthA (2)***	***fpg (2)***, *mag*, *mutY*, *nth*, *ung*, *xthA*
**Mismatch Repair**^b^**(MMR)**	*mutS*, *mutL*	*mutS*, *mutL*	*mutS*, *mutL*
**Direct Repair**	*alkB*, *phr*, *ogt*	*alkB*, *phr*, *ogt*,	*alkB*, *ogt*
**Recombination Repair**	*recA*, *recBCD*, *recF*, *recG*, *recJ*, *recN*, *recO*, *recR*, *recQ*, *ruvABC*, *sbcB*	*recA*, *recBCD*, *recF*, *recG*, *recJ*, *recN*, *recO*, *recR*, *recQ*, *ruvABC*, *sbcB*	*recA*, *recBCD*, *recF*, *recG*, *recJ*, *recN*, *recO*, *recR*, *recQ*, *ruvABC*, *sbcB*
**Other DNA Repair related genes**	*ada*^a^, ***lexA(2)***, *dinB*, *ligA*^c^, ***ligB***^c ^***(2)***	*ada*^a^, ***lexA(2)***, *dinB*, *ligA*^c^, ***ligB***^c ^***(3)***, *umuDC*	*lexA*, *ligA*^c^

Genome sizes are indicated within brackets. The DNA repair genes identified in the genome of the three organisms are shown. Those present in more than one copy due to duplications are marked in bold (numbers in parenthesis). ^a^*alkA *and *ada *regulatory domain genes are fused in these bacteria. ^b^*mutH*, found in *E. coli*, is not present in these 3 genomes. ^c ^*ligA *corresponds to the NAD-dependent ligase and *ligB *to the ATP-dependent ligase (see text).

**Figure 1:**
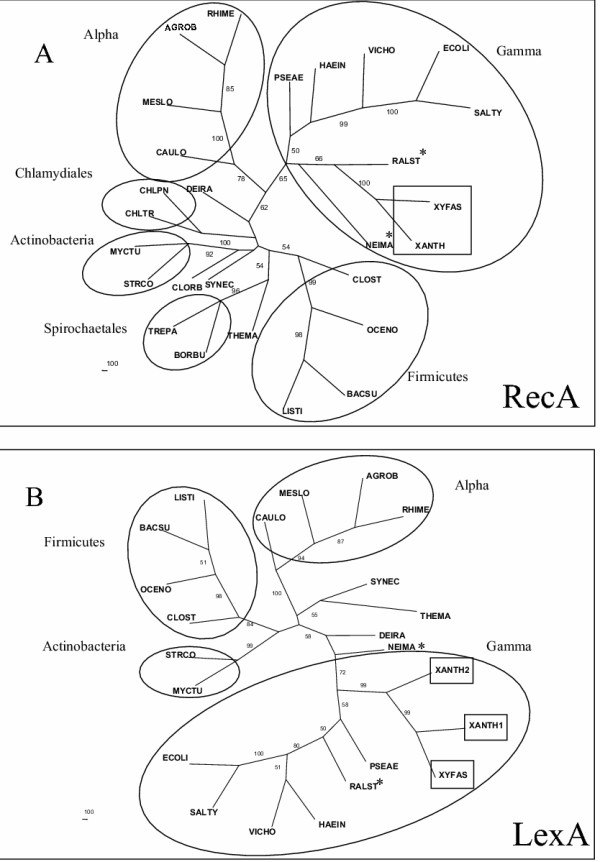
**Consensus unrooted trees generated by the Neighbor-Joining distance method for the RecA (A) and for the LexA proteins (B). **The circles highlight the main groups of bacteria. The symbol * indicates the beta proteobacteria. Some groups of bacteria included in (A) which are absent in the other trees: Spirochaetales: TREPA: *Treponema pallidum *(gi| 7443874), BORBU: *Borrelia burgdorferi *(gi| 15594476); Chlamydiales: CHLTR *Chlamydia trachomatis *(gi| 7443880), CHLPN: *Chlamydophila pneumonia*e CWL029 (gi| 7443883). The homologs of *X. axonopodis *and *X. fastidiosa *are indicated inside the square boxes.

The RecA protein is almost universal among bacteria and is a clear example of phylogeny that follows 16S rRNA gene evolution. In fact, this gene is always unique in all genomes analyzed and has been proposed as an alternative molecule to be used in systematic studies of Bacteria [2]. Figure 1A shows the phylogenetic tree generated for this protein, and confirms the distinction of the main groups of bacteria, such as Firmicutes, Chlamydiales, Spirochaetales and Proteobacteria. Among the proteobacteria, the Xanthomonadales branch is independently positioned at the root of gamma subdivision. It is important to note that although this seems to be consistent with the classification of these bacteria as part of gamma proteobacteria, very often the trees, from these and other proteins (see below), place the Xanthomonadales in the same branch as beta proteobacteria (*Neisseria *and *Ralstonia*).

In *E. coli*, the RecA protein has been shown to participate in recombinational repair, as well as in the control of a set of physiological changes to DNA damage, known as SOS response. The induction of this regulon stems from the co-protease activity of the RecA protein, which cleaves a repressor protein, denominated LexA, allowing the expression of the set of SOS genes. The presence of *lexA *and *recA *homologs in the Xanthomonadales indicates that these bacteria also present a SOS regulon. However, in *Xanthomonas *genomes, there are two copies of the *lexA *gene. The phylogeny of LexA proteins is presented in Figure 1B. The general topology of this tree indicates that LexA evolution follows a similar pattern to RecA, although the *lexA *gene is not found in all groups of bacteria. The products of the two copies of the *Xanthomonas lexA *paralogs are positioned close together in the same branch of Xanthomonadales, indicating a recent duplication of such a gene. The fact that they had branched before *Xylella*'s single *lexA *divergence indicates that this bacterium may have lost the duplication. Gene losses are expected to be extensive in *Xylella*, in agreement with this bacterium having a more specialized parasitic way of life [10]. The duplication of *lexA *in *Xanthomonas *points to a highly controlled SOS regulon, that may be important for fine-tuning the bacterial responses to stress induced in environmental changes. Indeed, functional characterization of *lexA1 *in *X. campestris *has been performed by gene disruption, and the data indicate this protein controls the expression of the *recA *gene, as expected for the SOS regulatory circuit [11]. The function of the second paralog, however, remains to be elucidated. Recent report indicates that the disruption of the *lexA *gene in *Deinococcus radiodurans *does not change the level of RecA expression, suggesting that LexA protein may be related to functions other than controlling the SOS regulon in that organism [12].

The nucleotide excision repair (NER) pathway is one of the most important, versatile and conserved systems of DNA damage removal in bacteria. It recognizes damages, which cause significant helix distortions in the DNA molecule, these being excised as an oligonucleotide by several enzymes that act as helicases and endonucleases. The main proteins that participate in such a pathway are known as UvrA, UvrB and UvrC, which, in sequential steps, interact with one another, recognize the damaged strand (UvrA), open the double helix (UvrB), and cleave DNA (UvrC) at both sides, few nucleotides away from the lesion. Subsequently, the oligonucleotide containing the damage is removed by a DNA helicase known as UvrD. The *uvrB *gene from *X. campestris *has been cloned and it was shown to participate in the resistance of these bacteria after UV irradiation [13]. The NER proteins are very well conserved and universal among bacteria. A complete set of orthologs of these genes is also found in a few species of the group Euryarchaeote: *Methanothermobacter thermautotrophicus*, *Methanosarcina sp and Halobacterium *sp. NRC-1 [14]. Other archaea may have a different unknown NER system [15].

**Figure 2:**
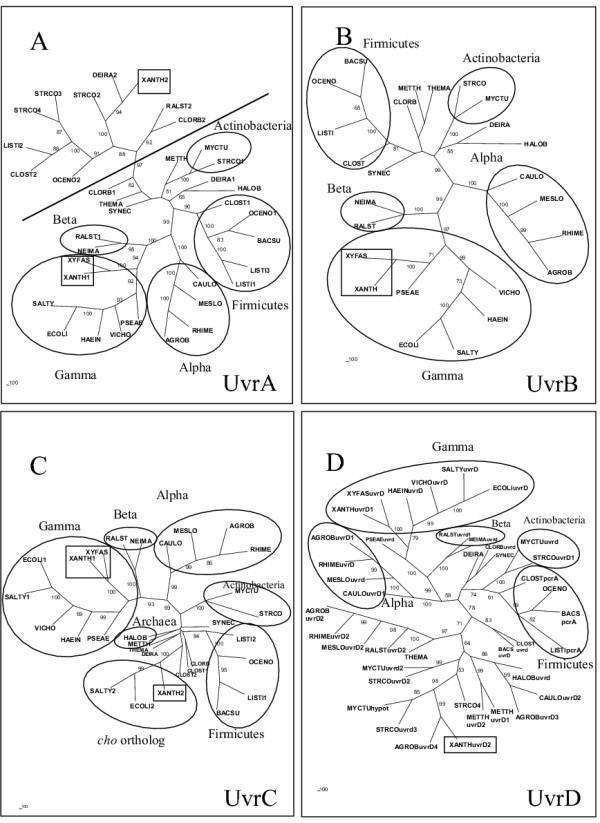
**Consensus unrooted trees generated by the Neighbor-Joining distance method for the UvrA (A), UvrB (B), UvrC (C) proteins and for the UvrD helicase family (D). **The numbers in front of organism names indicate the number of members of this gene family in the corresponding organism. The circles highlight the main groups of bacteria. Inside the square box, the homologs of *X. axonopodis *and *X. fastidiosa. *In (A) there is a clear distinction between the two UvrA orthologs separated by the line. In the upper part of the figure are grouped the organisms containing the second UvrA homolog, for which no function in DNA repair has yet been assigned. In (D) the names of the genes are based on annotation available.

The genes *uvrA*, *uvrC *and *uvrD *are duplicated in *Xanthomonas *and the phylogenies of the proteins encoded by these orthologs, together with the single *uvrB*, are presented in figure 2. In general, the evolution of these proteins, particularly UvrB (figure 2B), follows a pattern similar to RecA, the NER protein of *Xanthomonas *being positioned in the same branch of the *Xylella*'s ortholog, close to gamma proteobacteria as expected for a vertical descent. However, for the duplications, the patterns are different and more complex. The second ortholog of *uvrA *in *X. axonopodis *is phylogenetically closer to the also duplicated genes found in several other bacteria (figure 2A), thus indicating an old duplication event in evolution. The absence of this ortholog in most of the proteobacteria could be due to extensive gene loss. However, the phylogenetic proximity with unrelated organisms (including a duplication in *D. radiodurans*) points to an origin due to horizontal gene transfer from other bacteria. The functions of both *uvrA *orthologs are not necessarily related to DNA repair, since this activity can be provided by either one of them. It is relevant to mention that for the duplication found in *D. radiodurans*, White *et al *[16] have proposed a function related to the transport of damaged DNA out of the cell. This is due to the similarity of the UvrA protein with the ATP-binding subunit of a multifamily of genes involved in the transport across membranes, related to ABC transporters [17].

A similar pattern of evolution was observed for the phylogeny of UvrC protein (figure 2C). One of the two copies present in *X. axonopodis *is close to the *uvrC *orthologs from other gamma proteobacteria, following a vertical transmission. However, a protein with considerable similarity to the N-terminal of UvrC is found in both *Xanthomonas *species. These orthologs form an independent branch of the phylogenetic tree of UvrC, close to a heterogeneous group of gram-positive bacteria. The role of this second UvrC homolog of *E. coli *in DNA repair (named Cho protein, for Uvr***C ho***molog) has recently been investigated in detail, and was shown to have an endonuclease activity in damaged DNA [18]. This protein makes an incision only at the 3' side of the lesion, while the well-known UvrC was demonstrated to incise both sides of the lesion *in vitro *[19]. Cho may backup UvrC in the repair process of certain kinds of obstructive damage, possibly broadening the repair capacity of the excision pathway in these bacteria [20]. The origin of such orthologs is not clear, and although other bacteria may present protein domains that have some similarity to Cho endonuclease, known complete Cho homologs are limited to the *Azotobacter vinelandii*, *Escherichia coli*, *Salmonella typhimurium*, *Shiguella flexneri*, and *Xanthomonas*. Curiously, in Beta proteobacteria, *Ralstonia metallidurans *and *Chromobacterium violaceum*, an endonuclease domain similar to Cho appears as a C-terminal fusion with a putative 3'-exonuclease, corresponding to the epsilon subunit of DNA polymerase III. A similar fusion protein was found in *Mycobacterium tuberculosis*, and an interesting coordinated action of these two activities (endonuclease and exonuclease) was proposed as a new mechanism of DNA repair [20]. A former duplication could explain the origin of *cho *in these bacteria, but, again, one would have to concede extensive gene loss in other species. Once more, horizontal transfer events, involving part of the *uvrC *gene (the one that encodes the N-terminal) to some gamma proteobacteria, could explain the limited occurrence of *cho *only in these bacteria. Moreover, some organisms present a duplication of the complete *uvrC *gene (*Clostridium acetobutylicum *and *Listeria*). The proteins encoded by these paralogs are closely positioned in the tree, thus indicating a recent origin for them, and, possibly, functional redundancy.

**Figure 3:**
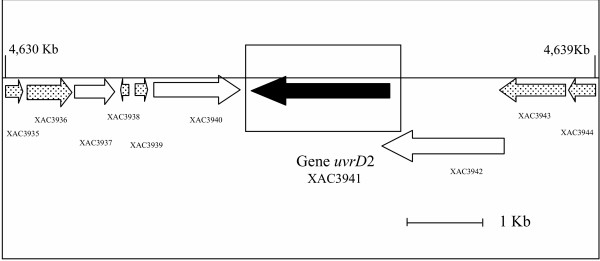
**Location vicinity of the *uvrD *homolog (*uvrD*2) gene in the genome of *Xanthomonas axonopodis *pv. citri. **A bold arrow in the square box represents the ORF of this gene. The dotted arrows on each side represent transposon related proteins and the white ones represent hypothetical proteins. The numbers of Kb indicate the position at the genome. The accession numbers of the proteins corresponding to the genes shown in the figure are: gi| 21244654 (XAC3935), gi| 21244655 (XAC3936), gi| 21244656 (XAC3937), gi| 21244657 (XAC3938), gi| 21244658 (XAC3939), gi| 21244659 (XAC3940), gi| 21244660 (XAC3941), gi| 21244661 (XAC3942), gi| 21244662 (XAC3943), gi| 21244663 (XAC3944)

The *uvr*D gene is part of a DNA helicase family, which includes the *pcr*A and *rep *genes [21]. The *uvrD *gene participates in the removal of damaged DNA strand, after the incision steps of NER or DNA mismatch repair have occurred. The evolution tree of these proteins is presented in figure 2D. Similar to *uvrA *and *uvrC*, there are two orthologs for these genes in the *X. axonopodis *genome. One of the orthologs encoded by the *uvrD *gene is closer to the gamma proteobacteria, branching with *X. fastidiosa*, following a typical vertical inheritance, while the second is similar to proteins from other prokaryotes, including Archaea and Bacteria, mainly from the firmicutes and alpha proteobacteria groups. The second ortholog of this gene from *Sinorhizobium meliloti *is found in plasmid DNA for which an alien origin is suggested, due to its lower GC content [22]. Additional analysis of the *uvrD *duplication in the *X. axonopodis *genome indicates that it is located close to transposon-related genes (Figure 3), the G+C content within this region (58.2%) being low when compared to what is found in the rest of the genome (average 64.7%). Moreover, the closest ortholog of this gene is also found in a plasmid of *Agrobacterium tumefaciens *(figure 2D). These data are compatible with a recent LGT event for this gene. The absence of *uvrD *duplication in the genome of *X. campestris *gives further support to the LGT hypothesis, indicating that it has been recently acquired in the *X. axonopodis *genome, possibly by means of plasmid transfer and/or transposon insertion.

Base excision repair (BER) protects genetic material from a wide range of DNA damaging agents [23]. The plasticity of this repair pathway is given by the presence of several different glycosylases, lesion recognition proteins that catalyze the first step in BER. The three organisms present similar sets of glycosylases, but *X. fastidiosa *bears two identical copies of *fpg*, probably due to its close proximity to the also duplicated rRNA genes [8]. This duplication may also provide to this bacterium, an enhanced protection against oxidative DNA damage. The presence of duplicated *xthA *homologs (apurinic/apyrimidinic endonuclease) in both *Xanthomonas *is another remarkable feature of BER in this group. Phylogenetic trees generated for this protein family show these orthologs located in different branches, indicating that the duplication is not a recent event. However, a miscellaneous branching pattern of the tree obtained for the different bacterial groups, results difficult to track the evolution of these genes (data not shown).

**Figure 4:**
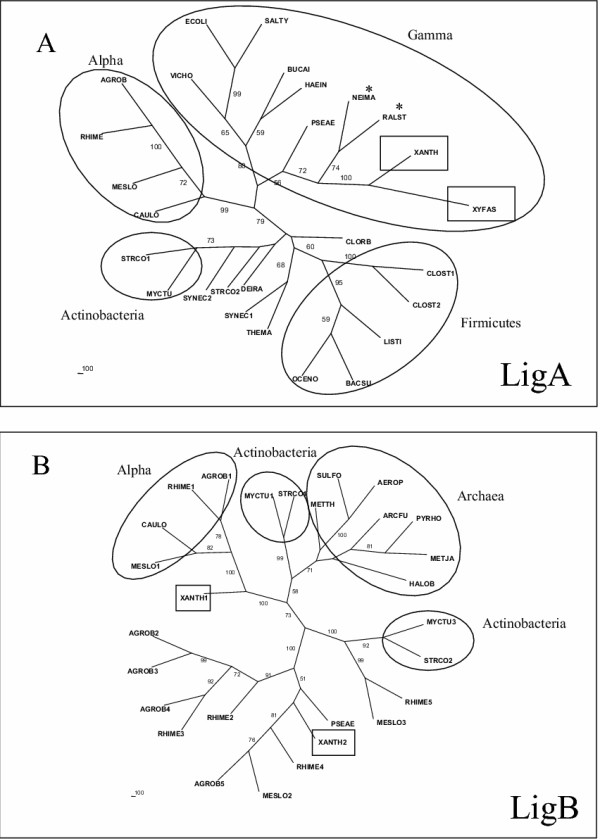
**Consensus unrooted trees generated by the Neighbor-Joining distance method for the NAD+ dependent (A) and ATP-dependent DNA ligases (B). **The circles highlight the main groups of bacteria. The homologs in Xanthomonadales are in square boxes. The symbol * indicates the beta proteobacteria. Some copies of homologs were excluded from this analysis given the low similarity among the DNA ligase ATP-dependent.

The DNA ligases catalyze the joining of breaks in the phosphodiester backbone of the DNA molecule and, thus, play an essential role in several processes of DNA repair, replication and recombination. These enzymes are evolutionary related, although two distinct families of DNA ligases are found: one that is typical for Bacteria, using NAD+ as cofactor, and a second that is typical of Eukaryotes and Archaea, but using ATP as cofactor (reviewed by Wilkinson *et al *[24]). As for all bacteria, only single copies of the *ligA *gene, encoding the NAD-dependent DNA ligase, is found in the *Xanthomonas *and *Xylella *genomes. The phylogeny for these proteins is presented in figure 4A, and it clearly follows a vertical descent, with the Xanthomonadales branch close to gamma and beta proteobacteria. However, as described for other bacteria, both *Xanthomonas *genomes present extra copies of putative ATP-dependent DNA ligases (two in *X. axonopodis *and three in *X. campestris*). The phylogenetic tree of ATP-dependent DNA ligases is shown in figure 4B. There is a clear independent branch, where most of the archaeal ATP-dependent DNA ligases are found, except for the orthologs observed in *Mycobacterium tuberculosis *and *Streptomyces coelicolor*. The other bacterial orthologs branch independently, wherein alpha proteobacteria predominate. A curious observation is the high and variable number of ATP DNA-ligases within the genomes of plant symbiontes, especially in the alpha proteobacteria (six in *Agrobacterium tumefasciens*, nine in *Sinorhizobium meliloti *and eleven in *Mesorhizobium loti*). It is possible that these *X. axonopodis *genes may have arisen from recent duplications, soon after gene introduction in alpha proteobacteria, probably by means of LGT from Archaea. The fact that these bacteria inhabit a common niche in plants could facilitate mutual gene exchange. However, the function of such ligases in these bacteria is unknown. As the NAD (+)-dependent ligase is present in all bacterial genomes, these Archaea-related ligase orthologs, present in certain bacteria, seem to be redundant, their roles in cell metabolism remaining a puzzle. Since NAD^+^-dependent DNA ligases are typically eubacterial, and cannot be replaced [24], the presence of additional ATP-dependent ligases is an unsolved question. Several lines of evidence indicate that the ligation reactions can be processed with different fidelity depending on the enzyme. Bacterial NAD^+^-dependent DNA ligases appear to perform more accurate ligation reactions, with few mispaired nucleotides being allowed in the DNA extremities [25]. NAD^+^-dependent DNA ligase from *Thermus *species exhibit enhanced mismatched ligations under certain conditions but catalyze reactions with 1–2 orders of magnitude more discriminative towards correct nucleotide matches than the ATP-dependent DNA ligase from T4-phage [26]. Working with the ATP-dependent DNA ligase from the hyperthermophilic archaeon, *Thermococcus kodakaraensis*, Nakatani *et al *[27] found that the enzyme could seal substrates with mismatched base-pairing at the 5' end of the nick, but did not show activity towards the 3' mismatched substrates.

Therefore, the presence of ATP-dependent DNA ligases in certain bacteria could be connected to the ligation of DNA breaks under several contexts that would generate genetic variability, with possible evolutionary advantages. In the alpha-proteobacteria group, a non homologous end joining appears to be important for the integration of inserting elements in the genome of host plants, as occurs with *Agrobacterium sp *T-DNA [28] or for the amplification of *Rhizobium *species *amplicons *present in pNGR234 plasmid [29]. In *Xanthomonas *the presence of the ATP-dependent DNA ligase could also be linked to the elevated number of transposable elements [9]. However, the association of this type of DNA ligase with specific processes that lead to genetic variability, as proposed above, is still under investigation.

Some other features of the DNA repair genes in Xanthomonadales are interesting to mention (Table 1). The absence of a putative photolyase gene (*phr*) in *X. fastidiosa*, while present in both *Xanthomonas*, may be related to its limited habitat within the plant xylem or inside the insect vector [8]. It is also remarkable that only *X. campestris *bears the *umuC *and *umuD *genes, which encode the DNA polymerase V (UmuD'_2_C), related to translesion synthesis [30]. In fact, both genes are located at the genome close to bacteriophage related genes [9], suggesting a recent acquisition by LGT, in a similar manner to *X. axonopodis uvrD2 *gene.

## Conclusions

Since the genomes of *Xanthomonas *(5.1 Mbp) are much larger than that of *X. fastidiosa *(2.7 Mbp), it was not a surprise to find more duplicated DNA repair genes in the former bacteria. Mechanisms for the protection of genetic information may reflect how the organism deals with stress and a hostile environment. Thus, the increased number of DNA repair genes in *Xanthomonas *may be due to the fact that these bacteria have a more variable habitat when compared to *Xylella*, which lives most of the time inside its hosts as a parasite. In this work, DNA repair genes, which appeared duplicated in the genomes, were analyzed focusing on their evolution, although it should be pointed out that their functions in vivo remain to be investigated. The duplicated genes found in *Xanthomonas *have close orthologs in other bacteria. As *Xanthomonas *are very closely related to *Xylella*, it is thus possible that, in these cases, duplications arose before the split *Xanthomonas*-*Xylella*, and were lost in the latter.

For the genes investigated, evolution patterns indicate that duplicated genes may result mainly from relatively ancient origins. Phylogenetic errors of construction, such as long branch attraction effect, cannot be completely excluded, although the presence of close orthologs reinforce the trees generated. Moreover, the duplications are normally positioned near to orthologs also found in the genomes of some distantly related bacteria. A clear exception is the most likely recent duplication found for the *lexA *paralogs of *Xanthomonas*, but this seems to be an unusual example. The possibility that gene duplications would have occurred in an early common ancestor, followed by gene loss in most other bacteria, cannot be the only explanation for many of the genes analyzed. Therefore, horizontal gene transfer among different bacteria possibly originated some of these paralogs. A clear example of recent LGT is the *uvrD *duplication, which is often found associated with DNA mobile elements. An origin from other life kingdoms may also have occurred, as for the ATP dependent DNA ligase, a common ligase in Archaea, which may have been acquired and were established in certain bacterial genomes, including *Xanthomonas*. The LGT, more than any other genetic process, makes possible faster ecological changes with the immediate incorporation of a gene or group of genes [31]. Eventually, organisms may acquire pathogenic features by LGT events [32,33]. In fact, a more efficient DNA repair system to protect genetic information would provide pathogenic organisms with tools to respond to stress caused in the host-pathogen interaction [10].

The new genes acquired by lateral gene exchange are expected to be maintained when they provide a selective advantage to the recipient cell [5]. For the DNA repair genes investigated in this study, most resulted in redundancy, pointing to function diversification among the orthologs. This seems to be the case of the *uvrC *homolog (*cho*), which has been found to have a complementary function in *E. coli *[18-20]. The fact that the closest orthologs, such as ATP dependent DNA ligases, are also observed in other bacteria that interact with plants may indicate both that they may play important roles in this interaction and in their necessity to adapt to the host. A common niche could also favor genetic exchange among these bacteria and would provide for the possibility of their sharing similar molecular mechanisms.

In *Mycobacterium tuberculosis *some DNA repair genes are induced by DNA damage, independently of the RecA protein [34]. Among them are orthologs of *uvr*A, *uvrD *and *ligB*, which are duplicated in Xanthomonas. This novel mechanism present in *M. tuberculosis *may also occur in *Xanthomonas *and support the idea that these duplications are also required protecting the genome against damage. Curiously, the duplicated genes found in *Xanthomonas *do not replace the orthologs that present vertical inheritance, similar to 16S rRNA. This reinforces the idea that they may complement the known DNA repair mechanisms with other different functions. The search for such novel functions for these genes may not only improve our knowledge on how cells protect their genomes against DNA damage, but also about how DNA repair processes evolve in bacteria.

## Methods

Sequences of DNA repair-related proteins were obtained at the National Center of Biotechnology Information GenBank database (35). The list of organisms, with abbreviations used and proteins analyzed, is shown in Table 2. An expanded version, containing all accession numbers of the genes employed in phylogenetic analyses, is shown in Table S1 (see additional file 1). The analysis of *Xanthomonas *was performed comparing the two different genomes of the species *X. axonopodis *and *X. campestris. *As most of their genes are very similar, only *X. axonopodis *homologs are shown, differences being described in the text. Protein sequences from complete genomes (Bacteria and Archaea) were aligned using the ClustalX multiple sequence alignment program [36] with manual adjustment with Genedoc (v2.6.02). Only unambiguously aligned positions (excluding poorly conserved and gap regions) were used in phylogenetic analysis. Phylogenetic trees were generated for each group of protein homolog from sequence alignments using the Phylip program version 3.5 [37]. Parsimony analysis was conducted using the Protpars program, and distance methods were performed using the Neighbor-Joining method in Phylip, with the distance PAM matrix model [38]. Bootstrap support (resampled 1,000 times) was calculated, and strict consensus trees constructed. Only bootstrap values greater than 50% are shown. Similar topologies were found for both algorithms employed, and only Neighbor-Joining is displayed. The consensus trees so obtained were viewed through TreeView software [39]. The same set of prokaryote species was used in all analyses, although few organisms were excluded from some trees, for simplification. The option for non-rooted trees aims at demonstrating only relationship among organisms without, however, linking ancestors and descendants.

**Table 2:** Presence of DNA repair genes investigated in this work.

	**Taxa**	Genes^b^						
		
Organisms (Abbreviation)^a^		*lex*A	*lig*	*rec*A	*uvr*A	*uvr*B	*uvr*C	*uvrD *family
*Aeropyrum pernix *(AEROP)	Archaea	-	1	-	-	-	-	-
*Archaeoglobus fulgidus *(ARCFU)	Archaea	-	2	-	-	-	-	-
*Halobacterium *(HALOB)	Archaea	-	1	-	1	1	1	1
*Methanobacterium thermoautotrophicum *(METTH)	Archaea	-	1	-	1	1	1	2
*Methanococcus jannaschii *(METJA)	Archaea	-	1	-	-	-	-	-
*Pyrococcus horikoshii *(PYHOR)	Archaea	-	1	-	-	-	-	-
*Sulfolobus solfataricus *(SULFO)	Archaea	-	1	-	-	-	-	-
*Mycobacterium tuberculosis H37 *(MYCTU)	Actinobacteria	1	4	1	1	1	1	3
*Streptomyces coelicolor A3(2) *(STRCO)	Actinobacteria	1	5	1	4	1	1	4
*Chlorobium tepidum TLS *(CLORB)	Chlorobi	-	1	1	2	1	1	1
*Bacillus subtilis *(BACSU)	Firmicutes	1	3	1	1	1	1	2
*Clostridium acetobutylicum *(CLOST)	Firmicutes	1	2	1	2	1	2	2
*Listeria innocua *(LISTI)	Firmicutes	1	1	1	3	1	2	1
*Oceanobacillus iheyensis *(OCENO)	Firmicutes	1	2	1	2	1	1	1
*Deinococcus radiodurans *(DEIRA)	Thermus/ Deinococcus	2	1	1	2	1	1	1
*Thermotoga marítima *(THEMA)	Thermotogae	1	1	1	1	1	1	1
*Synechocystis *(SYNEC)	Cyanobacteria	1	2	1	1	1	1	1
*Agrobacterium tumefaciens Cereon *(AGROB)	Alpha proteobacteria	1	7	1	1	1	1	2
*Caulobacter crescentus *(CAULO)	Alpha proteobacteria	1	2	1	1	1	1	2
*Mesorhizobium loti *(MESLO)	Alpha proteobacteria	1	12	1	1	1	1	2
*Sinorhizobium meliloti *(RHIME)	Alpha proteobacteria	1	10	1	1	1	1	2
*Neisseria meningitidis *Z2491 (NEIMA)	Beta proteobacteria	1	1	1	1	1	1	1
*Ralstonia solanacearum *(RALST)	Beta proteobacteria	1	1	1	2	1	1	2
*Buchnera sp *(BUCAI)	Gamma proteobacteria	-	1	-	-	-	-	-
*Escherichia coli. *(ECOLI)	Gamma proteobacteria	1	1	1	1	1	2	1
*Haemophilus influenzae *(HAEIN)	Gamma proteobacteria	1	1	1	1	1	1	1
*Pseudomonas aeruginosa *(PSEAE)	Gamma proteobacteria	1	2	1	1	1	1	1
*Salmonella typhimurium LT2 *(SALTY)	Gamma proteobacteria	1	1	1	1	1	2	1
*Vibrio cholerae *(VICHO)	Gamma proteobacteria	1	2	1	1	1	1	1
*Xanthomonas axonopodis *pv citri (XANTH)	Gamma proteobacteria	2	3	1	2	1	2	2
*Xylella fastidiosa *(XYFAS)	Gamma proteobacteria	1	1	1	1	1	1	1

a. The organisms are listed in alphabetic order within the taxa. b. The numbers indicate the amount of homologs.

## Authors' contributions

MM-P carried out the phylogenetic analyses and, together with RSG and CFMM, designed and conceived the ideas and the writing of the manuscript. CL gave substantial contribution on the possible involvement of ATP-dependent DNA ligases, when present in bacterial genomes. KML-B and KAA participated in the sequencing and DNA repair genes annotation in *Xanthomonas sp. *All authors read and approved the final manuscript.

## Supplementary Material

Additional File 1 Accession numbers for the genes indicated in the figures. Contains all accession numbers of the genes used in the phylogenetic analyses. Click here for file
