# Supplementary material for: Different patterns of evolution for duplicated DNA repair genes in bacteria of the Xanthomonadales group
Source: BMC Evol Biol. 2004 Aug 27;4:29. doi: 10.1186/1471-2148-4-29 (PMC518961; doi:10.1186/1471-2148-4-29)
Supplement: Additional File 1 — Accession numbers for the genes indicated in the figures. Contains all accession numbers of the genes used in the phylogenetic analyses. [file 1471-2148-4-29-S1.doc]

Table 1S – Accession numbers for the genes indicated in the figures.

| **Organisms (Abbreviation)** | Genes | | | | | | | |
| --- | --- | --- | --- | --- | --- | --- | --- | --- |
|  | ***lex*A** | ***ligA*** | ***ligB*** | ***rec*A** | ***uvr*A** | ***uvr*B** | ***uvr*C** | ***uvrD* family** |
| Aeropyrum pernix (AEROP) | - | - | gi|7447285 | - | - | - | - | - |
| ***Archaeoglobus fulgidus* (ARCFU)** | - | - | gi|11498231 | - | - | - | - | - |
| ***Halobacterium* (HALOB)** | - | - | gi|15790019 | - | gi|15791365 | gi|10581796 | gi|10581790 | gi|15791353 |
| ***Methanobacterium thermoautotrophicum* (METTH)** | - | - | gi|2494167 | - | gi|8134782 | gi|3122998 | gi|8134792 | gi|15678500  gi|15678539 |
| ***Methanococcus jannaschii* (METJA)** | - | - | gi|2494166 | - | - | - | - | - |
| ***Pyrococcus horikoshii* (PYHOR)** | - | - | gi|6225280 | - | - | - | - | - |
| ***Sulfolobus solfataricus* (SULFO)** | - | - | gi|13813323 | - | - | - | - | - |
| Mycobacterium tuberculosis7 (MYCTU) | gi|7448344 | gi|7674014 | gi|15610199  gi|15610867 | gi|12084113 | gi|15841093 | gi|13881302 | gi|7443943 | gi|15608089  gi|15610334  gi|15610337 |
| [Streptomyces coelicolor](http://www.ncbi.nlm.nih.gov/htbin-post/Taxonomy/wgetorg?id=1902) A3(2) (STRCO) | gi|7481782 | gi|7674017  gi|14495032 | gi|21219712  gi|21225001 | gi|21224115 | gi|21220443  gi|21219437  gi|21225013  gi|21224819 | gi|32141151 | gi|21220438 | gi|21223175  gi|21223557  gi|21223553  gi|21223552 |
| Chlorobium tepidum TLS (CLORB) | - | gi|21673293 |  | gi|21674742 | gi|21674507  gi|21673362 | gi|21674365 | gi|21674621 | gi|21673182 |
| ***Bacillus subtilis* (BACSU)** | gi|399378 | gi|7673987 |  | gi|2634066 | gi|3915204 | gi|3123297 | gi|137192 | gi|16077729  gi|16078247 |
| Clostridium acetobutylicum (CLOST) | gi|15895107 | gi|15895931  gi|15894478 |  | gi|15895091 | gi|15893794  gi|15894743 | gi|15893793 | gi|15893799  gi|15896707 | gi|15895932  gi|15894426 |
| ***Listeria innocua* (LISTI)** | gi|16800408 | gi|16800936 |  | gi|16800503 | gi|16801693  gi|16801222  gi|16800880 | gi|16801694 | gi|16800266  gi|16799913 | gi|16800937 |
| ***Oceanobacillus iheyensis* (OCENO)** | gi|23099124 | gi|23098215 |  | gi|23099079 | gi|38258782  gi|23097736 | gi|23099943 | gi|23099571 | gi|23098214 |
| ***Deinococcus radiodurans* (DEIRA)** | gi|15808003 | gi|7471821 | - | gi|6226893 | gi|15806772  gi|15807854 | gi|6460084 | gi|6116772 | gi|15806776 |
| ***Thermotoga marítima* (THEMA)** | gi|6225616 | gi|7674016 |  | gi|4982444 | gi|8134786 | gi|8134790 | gi|8134799 | gi|15643994 |
| ***Synechocystis* (SYNEC)** | gi|7443919 | gi|7674015  gi|7469299 |  | gi|2500096 | gi|16330044 | gi|3123003 | gi|3024785 | gi|16330097 |
| ***Agrobacterium tumefaciens* (AGROB*)*** | gi|15888721 | gi|15889366 | gi|15888181  gi|16119326  gi|16119904  gi|16119279  gi|15890358 | gi|15889174 | gi|17935412 | gi|15889291 | gi|15888466 | gi|25290780  gi|25290779  gi|15887382  gi|16119255 |
| ***Caulobacter crescentus* (CAULO)** | gi|16126145 | gi|13422903 | gi|13425358 | gi|13422391 | gi|16126828 | gi|13424613 | gi|13424497 | gi|16125775  gi|16127768 |
| ***Mesorhizobium loti* (MESLO)** | gi|13470826 | gi|13471538 | gi|13474572  gi|13473868  gi|13476676 | gi|14021092 | gi|13470922 | gi|13472362 | gi|14027316 | gi|13471509  gi|13476580 |
| ***Sinorhizobium meliloti* (RHIME)** | gi|15074543 | gi|15075186 | gi|15966661  gi|16264958  gi|16262677  gi|15966496  gi|16263759 | gi|15074733 | gi|15965310 | gi|15074835 | gi|15074103 | gi|15965897  gi|16263702 |
| ***Neisseria meningitidis* Z2491 (NEIMA)** | gi|15678006 | gi|11272942 | - | gi|9911105 | gi|15794105 | gi|9910889 | gi|11278017 | gi|15676157 |
| Ralstonia solanacearum (RALST) | gi|17546023 | gi|17546117 | - | gi|17545270 | gi|17545139  gi|17549215 | gi|17545730 | gi|17545790 | gi|17546954  gi|17549495 |
| ***Buchnera sp*** **( BUCAI)** | - | gi|11132251 | - | - | - | - | - | - |
| ***Escherichia coli****.***(ECOLI)** | gi|126224 | gi|2506362 | - | gi|132224 | gi|26250860 | gi|43284 | gi|1174919  gi|1788037 | gi|16131665 |
| ***Haemophilus influenzae* (HAEIN)** | gi|1074965 | gi|1169385 | - | gi|1172885 | gi|1174917 | gi|1174918 | gi|1573005 | gi|16273110 |
| ***Pseudomonas aeruginosa* (PSEAE)** | gi|585396 | gi|11348439 | gi|11351068 | gi|77683 | gi|15599430 | gi|12644339 | gi|11348491 | gi|15600636 |
| ***Salmonella typhimurium* *LT2*** **(SALTY)** | gi|417245 | gi|16761347 | - | gi|16761605 | gi|16767504 | gi|16759723 | gi|16765284  gi|16764660 | gi|16767221 |
| ***Vibrio cholerae*** **(VICHO)** | gi|11355622 | gi|11272938 | - | gi|11356058 | gi|15640421 | gi|15642773 | gi|11278014 | gi|15640220 |
| ***Xanthomonas axonopodis*  (XANTH)** | gi|21242488  gi|21241950 | gi|21242377 | gi|21242094  gi|21243148 | gi|21242489 | gi|38258806  gi|21241940 | gi|21243355 | gi|21242831  gi|21243291 | gi|21244868  gi|21244660 |
| ***Xylella fastidiosa*** **(XYFAS)** | gi|11362226 | gi|11360896 | - | gi|11387033 | gi|32130308| | gi|11278009 | gi|1127015 | gi|15836655 |
